# Short-Time Homomorphic Deconvolution (STHD): A Novel 2D Feature for Robust Indoor Direction of Arrival Estimation

**DOI:** 10.3390/s26020722

**Published:** 2026-01-21

**Authors:** Yeonseok Park, Jun-Hwa Kim

**Affiliations:** 1 Tech Innovation Group, KT Corporation, Seoul 03155, Republic of Korea; yeonseok.park@kt.com; 2 Department of Artificial Intelligence, Konyang University, Daejeon 35365, Republic of Korea

**Keywords:** indoor positioning, sound source localization, direction of arrival, short-time homomorphic deconvolution, time-of-flight, cepstral analysis, deep learning, Convolutional Neural Network, channel attention, sim-to-real, multi-channel audio

## Abstract

Accurate indoor positioning and navigation remain significant challenges, with audio sensor-based sound source localization emerging as a promising sensing modality. Conventional methods, often reliant on multi-channel processing or time-delay estimation techniques such as Generalized Cross-Correlation, encounter difficulties regarding computational complexity, hardware synchronization, and reverberant environments where time difference in arrival cues are masked. While machine learning approaches have shown potential, their performance depends heavily on the discriminative power of input features. This paper proposes a novel feature extraction method named Short-Time Homomorphic Deconvolution, which transforms multi-channel audio signals into a 2D Time × Time-of-Flight representation. Unlike prior 1D methods, this feature effectively captures the temporal evolution and stability of time-of-flight differences between microphone pairs, offering a rich and robust input for deep learning models. We validate this feature using a lightweight Convolutional Neural Network integrated with a dual-stage channel attention mechanism, designed to prioritize reliable spatial cues. The system was trained on a large-scale dataset generated via simulations and rigorously tested using real-world data acquired in an ISO-certified anechoic chamber. Experimental results demonstrate that the proposed model achieves precise Direction of Arrival estimation with a Mean Absolute Error of 1.99 degrees in real-world scenarios. Notably, the system exhibits remarkable consistency between simulation and physical experiments, proving its effectiveness for robust indoor navigation and positioning systems.

## 1. Introduction

Indoor Positioning Systems (IPS) are crucial for a wide range of applications, from robotic navigation and asset tracking to smart home assistants and emergency response [1]. While various technologies such as Wi-Fi, Ultra-Wideband (UWB), and Inertial Measurement Units (IMUs) are commonly used, audio sensor-based Sound Source Localization (SSL) offers a passive, low-cost, and versatile sensing solution [2,3]. This technology achieves indoor localization by estimating the Direction of Arrival (DoA) of specific acoustic signatures, such as communication signals broadcast by mobile devices. SSL fills a critical gap by enabling non-line-of-sight (NLOS) detection, providing vital context for indoor navigation and situational awareness where other sensors may fail.

Reverberation poses the primary challenge for audio-based SSL in indoor environments. Conventional algorithms that estimate the Time Difference in Arrival (TDOA) between microphone pairs, such as the Generalized Cross-Correlation with Phase Transform (GCC-PHAT) [4], degrade severely as noise and reflections create spurious peaks, masking the true direct-path signal [5]. Although methods like beamforming [6] offer spatial filtering, they typically require a large, precisely synchronized array and significant computational power.

Deep learning models, particularly Convolutional Neural Networks (CNNs), have emerged as a promising solution to address these limitations [7]. However, the efficacy of these approaches hinges primarily on the discriminative power of the input features. Concurrently, a separate line of research has focused on simplifying hardware complexity. Our previous works explored this domain, starting with monaural (single-microphone) systems using Homomorphic Deconvolution (HD) to estimate Time-of-Flight (ToF) from reflections [8]. This research established HD as a viable tool for ToF extraction and evolved into Single-Channel Multiple-Receiver (SCMR) systems. These SCMR systems used an analog adder to combine microphone signals, drastically reducing hardware complexity, and paired 1D HD features with machine learning models (e.g., Linear Regression [9], GPR [10], NN Regression [11]) to estimate the Angle of Arrival (AoA).

A fundamental limitation of these prior HD-based methods is their reliance on 1D features. Whether using parametric coefficients [12] or a 1D cepstral vector, these features represent the average ToF over the signal’s entire duration. This temporal averaging masks transient details; a brief noise burst and a stable sound source might produce similar 1D features, confusing a classifier.

**Figure 1:**
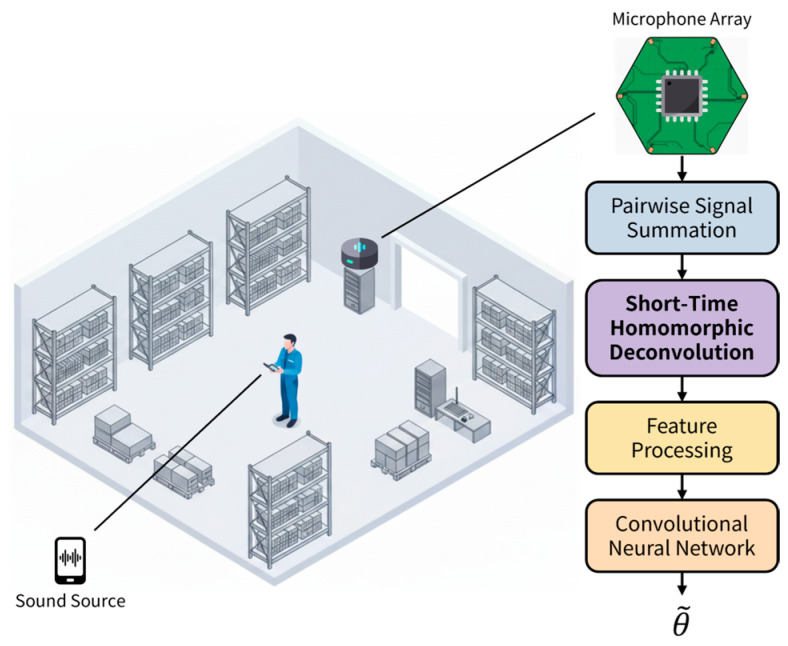
Overview of the proposed indoor sound source localization system. Multi-channel acoustic signals captured by a microphone array are processed through pairwise summation and Short-Time Homomorphic Deconvolution (STHD) to generate 2D feature representations, which are then fed into a Convolutional Neural Network (CNN) regressor to estimate the direction of arrival ($\tilde{\theta }$).

In this paper, we propose a significant advancement: Short-Time Homomorphic Deconvolution (STHD). Inspired by the utility of spectrograms over standard Fast Fourier Transforms (FFT), STHD represents time-varying ToF information. Unlike previous methods that compute ToF estimation as a single 1D vector, our approach generates a 2D feature map (Time × ToF), explicitly capturing the temporal dynamics and stability of the spatial cues. This rich, 2D representation is ideally suited for modern CNNs (Figure 1).

The main contributions of this paper are:

- A novel audio feature, STHD, that generates a 2D (Time × ToF) representation from microphone pairs for robust SSL, distinguishing it from prior 1D cepstral or parametric coefficient features [8,9,10,11,12].
- A high-quality, large-scale, simulation-based multi-channel audio dataset generated to ensure robust Sim-to-Real generalization.
- Comprehensive validation of the proposed STHD feature using a custom lightweight CNN architecture integrated with a dual-stage channel attention mechanism (Both-CNN), demonstrating its high discriminative power and suitability for modern deep learning models.

The remainder of this paper is organized as follows: Section 2 reviews related works. Section 3 details the STHD algorithm. Section 4 describes the methodology for validation. Section 5 and Section 6 present the simulation and experimental results, respectively. Section 7 concludes the paper.

## 2. Related Works

Sound Source Localization (SSL) has emerged as a promising sensing modality for indoor positioning systems, offering a passive and ubiquitous solution utilizing standard microphone arrays [13]. However, achieving robust localization in reverberant indoor environments remains a significant challenge. Traditional approaches largely depend on multi-channel signal processing techniques. Methods such as delay-and-sum beamforming [6] and high-resolution algorithms like MUltiple SIgnal Classification (MUSIC) [14] provide spatial filtering but entail high computational costs. The most widely adopted alternative, Time Difference in Arrival (TDOA) estimation via Generalized Cross-Correlation with Phase Transform (GCC-PHAT) [4], is computationally efficient but notoriously vulnerable to noise and reverberation [15], where reflections often mask the true direct-path signal.

Consequently, machine learning approaches have gained traction to overcome these limitations. Early data-driven methods utilized features such as Inter-channel Phase Difference (IPD) or spectrograms as inputs to Convolutional Neural Networks (CNNs) or Recurrent Neural Networks (RNNs) [16,17,18,19,20]. While these methods demonstrate improved robustness against noise, they often require complex model architectures to disentangle mixed spatial cues from spectro-temporal data. A distinct line of research, forming the foundation of this work, leverages cepstral analysis for Time-of-Flight (ToF) estimation. Homomorphic Deconvolution (HD) was initially explored for monaural SSL [8] and later extended to parametric HD [12] and simplified Single-Channel Multi-Receiver (SCMR) setups [9,10,11]. These studies demonstrated that 1D HD-based features could effectively map to an Angle of Arrival (AoA). However, these 1D representations suffer from temporal averaging, losing transient details crucial for distinguishing direct paths from reflections. In contrast, the proposed 2D STHD framework prevents this loss by preserving the time-axis information, thereby enabling the explicit capture of dynamic signal evolution that is otherwise obliterated by global averaging.

In parallel, attention mechanisms have been widely adopted to enhance feature representation within deep learning architectures. Specifically, the Squeeze-and-Excitation (SE) block, originally introduced for image classification CNNs to model channel-wise dependencies explicitly [21], adaptively recalibrates channel feature responses. By learning the global information of each channel, it emphasizes informative features while suppressing less useful ones. This mechanism has been successfully adapted to the audio domain for tasks such as speaker verification [22,23]. In the context of this study, the SE block is employed to learn the interdependencies among the 15 microphone pair channels, allowing the network to dynamically prioritize reliable pairs containing strong direct-path information while suppressing those corrupted by environmental noise or ambiguity.

## 3. Short-Time Homomorphic Deconvolution (STHD)

Traditional Homomorphic Deconvolution (HD) for Time-of-Flight (ToF) estimation [8] models the combined signal y[n] from a microphone pair as the convolution of the source signal x[n] and the channel impulse response h[n] (i.e., y[n] = x[n]∗h[n]). While effective for isolating h[n] to estimate ToF, Conventional HD relies on a global Fast Fourier Transform (FFT) applied to the entire signal block. This results in a 1D cepstral vector that represents the average ToF characteristics over the signal’s duration, inevitably masking transient details and losing information regarding the temporal stability of the sound source.

To overcome this limitation, we propose the Short-Time Homomorphic Deconvolution (STHD) algorithm. The fundamental innovation of STHD is the replacement of the initial global FFT with the Short-Time Fourier Transform (STFT). By mapping the signal to a 2D time-frequency representation, STHD enables the independent analysis of homomorphic features for each time frame, thereby capturing the temporal evolution of the ToF.

**Figure 2:**
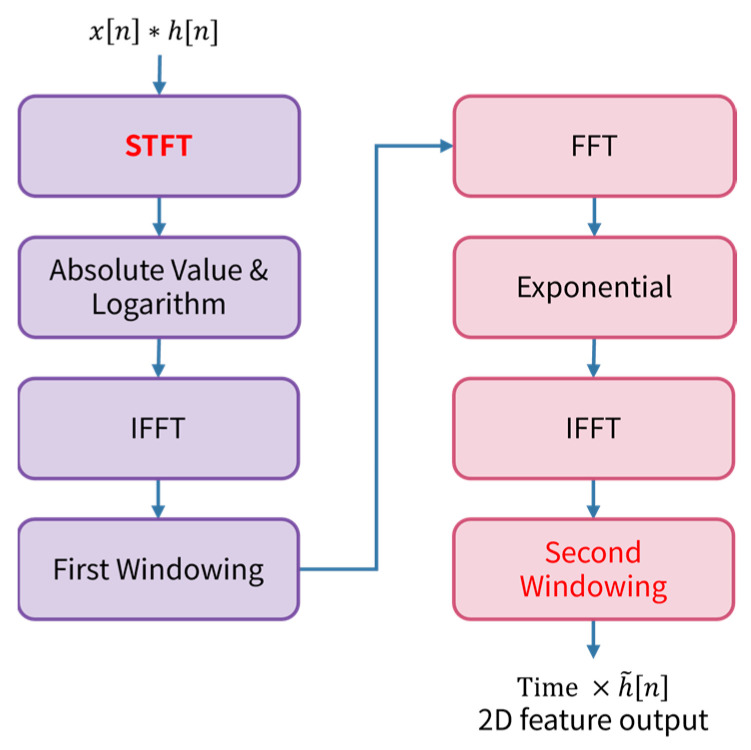
Block diagram of the proposed Short-Time Homomorphic Deconvolution (STHD) algorithm.

The overall process of the proposed STHD algorithm is illustrated in Figure 2. The procedure begins with the pairwise summation of microphone signals (e.g., $x_{1}+x_{2}$), followed by a sequential transformation to extract the 2D feature map. The specific steps are as follows:

- **STFT:** An STFT is applied to the paired signal $S_{ij}$, converting it into a complex spectrogram $Y\left(t,\omega \right)$ by analyzing the signal in short, overlapping frames.
- **Log-Magnitude:** The absolute value and logarithm are taken: $\log\left|Y\left(t,\omega \right)\right|$. This moves each time frame into the cepstral domain.
- **IFFT:** An Inverse FFT (IFFT) is applied to the log-spectrum independently for each time frame. This yields a time-varying cepstrum where the source components and channel components are separable [24].
- **First Windowing:** A windowing operation is applied to filter the cepstrum in each time frame, isolating the “rahmonics” (ToF components) $c_{h}\left(t,m\right)$ from the source components $c_{x}\left(t,\;m\right)$.
- **Inverse Process:** To recover the channel impulse response in the linear domain, the isolated cepstrum undergoes a forward FFT, an exponential operation (reversing the logarithm), and a final IFFT.
- **Second Windowing:** A final windowing operation is applied across the ToF axis to remove irrelevant peak values and artifacts outside the physical delay range of the array.

The output of this algorithm is a 2D matrix rather than a 1D vector, providing a temporal footprint of the spatial information. The specific hyperparameters used for the STHD implementation, including STFT and FFT settings, are summarized in Table 1.

Consequently, based on these parameters, this feature map has dimensions of 256 (Time frames) × 512 (ToF bins) for the given signal segments. The ToF axis corresponds to the time delay measured in samples. The theoretical sample delay ($Sample_{delay}$) between two microphones separated by a distance $D_{mic}$ can be calculated using the sampling frequency ($F_{s}$) and the speed of sound (c):

(1) $$Sample_{delay}=round\left(\frac{F_{s}}{c}\times D_{mic}\right)$$

Given the system’s sampling rate of $F_{s}=\;48,000\;Hz$ and the speed of sound c ≈340 m/s, the delay calculation is directly mapped to the ToF bin index. For instance, a microphone spacing of 0.1 m yields approximately 14.11 samples (rounded to 14), while a spacing of 0.05 m results in approximately 7.06 samples (rounded to 7).

**Figure 3:**
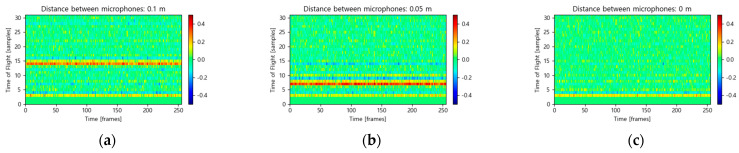
Visualization of representative 2D STHD feature maps for different microphone spacings. The color scale indicates the amplitude of the STHD coefficients, with red representing high positive peaks. (**a**) 0.1 m spacing showing a ridge at 14 samples. (**b**) 0.05 m spacing showing a ridge at 7 samples. (**c**) 0 m spacing showing no ridge in the search region due to artifact removal at zero lag.

Figure 3 visually demonstrates the geometric consistency of the STHD feature maps for three specific microphone spacings (0.1 m, 0.05 m, and 0 m), assuming a sound source at 0°. As predicted by the theoretical calculations, the feature maps display stable, high-amplitude ridges along the time axis (*x*-axis) at specific ToF indices. For the 0.1 m spacing (Figure 3a), a distinct energy ridge is centered precisely at the 14th sample index, matching the physical delay. Similarly, the 0.05 m spacing (Figure 3b) shows a clear ridge at the 7th sample index, validating the feature’s resolution even for smaller baselines. In contrast, for the 0 m spacing (Figure 3c), which represents pairs perpendicular to the source direction, no significant ridge is observed in the valid search range. The zero-lag component, corresponding to the direct path in this case, falls within the low-lag range (indices 0–3) that is explicitly suppressed by the windowing process to filter out artifacts. Unlike standard cross-correlation methods, which may fluctuate with ambient noise, the STHD feature maintains a consistent estimated trajectory over time, providing the CNN with robust and explicitly interpretable spatial cues.

## 4. Methodology

### 4.1. Receiver Configuration

**Figure 4:**
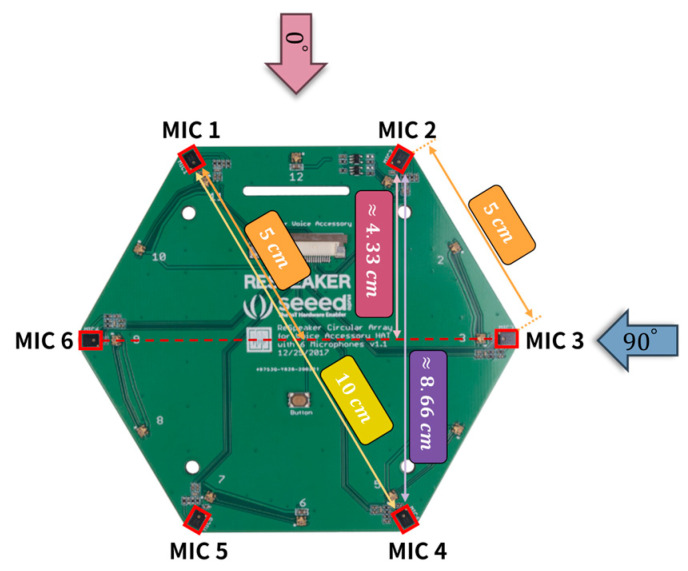
Configuration of the ReSpeaker 6-Mic Circular Array and geometric projection of baselines.

The implementation of the proposed STHD feature extraction framework utilizes a ReSpeaker 6-Mic Circular Array Kit (Seeed Studio, Shenzhen, China) for Raspberry Pi. As illustrated in Figure 4, this hardware features six microphones arranged in a uniform hexagonal geometry, effectively forming a Uniform Circular Array (UCA). The physical spacing between neighboring microphones is fixed at 5 cm, resulting in a maximum diametric baseline of 10 cm.

The six audio channels are combined into M=62 = 15 distinct pairs to capture rich spatial information. Localization relies fundamentally on the variation in the effective Time-of-Flight (ToF) difference for each pair, which is determined by the Angle of Arrival (AoA) of the incident sound. Under the far-field assumption, the effective baseline is defined as the projection of the physical distance onto the source direction vector.

For instance, assuming a sound source arrives from 0°, specific pairs, such as Mic 2 and Mic 3, exhibit an intermediate baseline. The projected distance is calculated trigonometrically as approximately 4.33 cm ($5\times \sin\left(60{}^{\circ}\right)$), which corresponds to a delay of approximately 6 samples at a 48 kHz sampling rate (c ≈340 m/s). In contrast, pairs with a wider geometry relative to the source, such as Mic 2 and Mic 4, yield an effective distance of approximately 8.66 cm, corresponding to a delay of 12 samples. Consequently, for any specific direction, the 15 microphone pairs generate a unique constellation of time delays, ranging from 0 cm (perpendicular incidence) to a maximum of 10 cm. This geometric diversity ensures that the STHD input feature contains a highly discriminative spatial signature for the subsequent regression model.

### 4.2. Feature Processing

**Figure 5:**
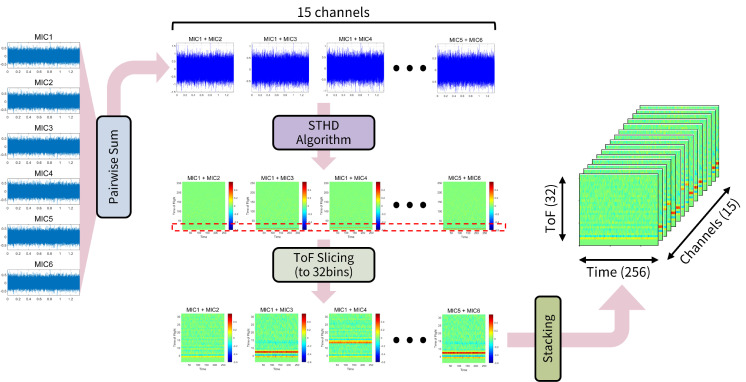
Overview of the feature processing pipeline. Pairwise signal summation generates 15 channel combinations processed by the STHD algorithm. The ToF axis is cropped to the first 32 bins (red dashed box) to isolate primary signal peaks, resulting in a stacked 256×32×15 input tensor.

The overall feature processing pipeline is illustrated in Figure 5. Optimization of the input feature map for the CNN involves adjusting data dimensions based on the physical characteristics of the microphone array and the properties of the STHD algorithm.

Regarding the channel dimension, the proposed method utilizes all 15 possible pairs derived from the 6 microphones rather than individual microphone features. Pairwise summation is applied to the array signals, followed by the STHD algorithm for each pair. The resulting feature maps are then stacked to form the channel depth. Consequently, based on the STFT hop size (256 samples) and FFT size (512 points) defined in Table 1, the final input data is structured as a 3D tensor with dimensions of 256×32×15 (Time frames × ToF bins × Channels). This multi-channel configuration enables the effective extraction of spatial features embedded within the correlations of all microphone pairs.

Subsequently, the Time-of-Flight (ToF) axis is sliced into 32 bins. Since the maximum inter-microphone distance is 10 cm, the maximum time delay corresponds to approximately 14 samples. Consequently, the primary STHD signal peaks are concentrated within this range. Cropping the ToF dimension to 32 bins (as indicated by the red dashed box in Figure 5) mitigates the risk of information loss near the boundaries during convolutional padding, ensuring critical features remain within the network’s receptive field.

Finally, per-sample standardization is applied to the stacked tensor to address potential weak spatial cues, such as those arising from short inter-microphone distances. Instead of using global dataset statistics, each sample is normalized individually by subtracting its own mean and dividing by its standard deviation. This individual normalization amplifies the relative signal strength within each sample, ensuring robust feature learning.

### 4.3. CNN Architecture for STHD

A custom lightweight 3-layer CNN architecture was designed to validate the discriminative power of the proposed 2D STHD features while ensuring computational efficiency for potential real-time applications. While Transformer-based architectures [25,26] utilizing self-attention mechanisms have recently gained popularity, their high computational complexity [27] poses significant challenges for deployment on resource-constrained edge devices typical of indoor positioning systems. In contrast, the integration of SE-Blocks within a lightweight CNN offers a highly efficient alternative, allowing the model to explicitly recalibrate channel-wise feature importance with negligible computational overhead compared to full self-attention layers. This dedicated network is optimized to process the specific dimensions of the feature tensor (256×32×15). Functioning as a regressor rather than a classifier, the model directly estimates the continuous Direction of Arrival (DoA) angles.

#### 4.3.1. Network Structure and Channel Attention

The proposed network is designed as a lightweight 3-layer CNN followed by a regression head. Implementation utilizes the channel-first format (C×H×W), where C, H, and W represent the Channels (microphone pairs), ToF bins, and Time frames, respectively. The backbone network consists of three convolutional blocks, each comprising a 3×3 2D convolution layer, Batch Normalization, ReLU activation, and Max Pooling. The pooling kernels were strategically chosen as (1,2), (1,4), and (2,4) to ensure that the time dimension (W) is compressed more aggressively than the ToF dimension (H), thereby prioritizing the preservation of critical spatial resolution within the STHD features.

A core component of our architecture is the Squeeze-and-Excitation (SE) Block [21]. The SE-Block is a channel attention mechanism that adaptively recalibrates channel-wise feature responses by modeling the interdependencies between feature channels. The block first performs a Squeeze operation (Global Average Pooling) on the feature map to aggregate global spatial information into a channel descriptor vector. Subsequently, an Excitation operation is executed using two fully connected (FC) layers with ReLU and Sigmoid activations, generating channel-wise scaling factors. These factors are explicitly multiplied with the original input feature map, effectively enhancing meaningful channels while suppressing less useful ones.

**Figure 6:**
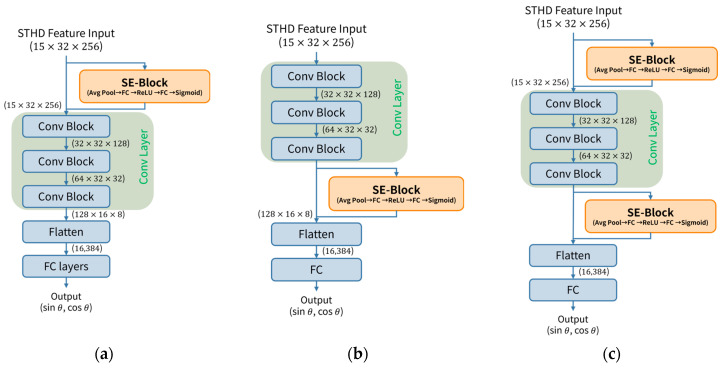
Comparison of the proposed STHD-CNN architectures. (**a**) Pre-CNN Attention: SE-Block applied to the input (15×32×256) to weight the microphone pair reliability. (**b**) Post-CNN Attention: SE-Block applied to high-level features after the final convolutional block. (**c**) Both-CNN Attention: A dual-stage approach integrating SE-Blocks at both the input and output stages.

Investigation into the optimal integration point for this channel attention mechanism led to the comparison of three distinct model configurations, as illustrated in Figure 6.

In the Pre-CNN Attention strategy (Figure 6a), the SE-Block is placed immediately at the input stage. Since the input channels directly correspond to the 15 explicit microphone pair combinations, this approach is defined as “STHD-Channel Attention”. This configuration allows the network to learn the reliability of each physical microphone pair before any complex convolutions occur, effectively amplifying reliable STHD maps while suppressing noisy ones.

Conversely, the Post-CNN Attention strategy (Figure 6b) places the SE-Block after the final convolutional block. Referred to as “High-Level Feature Attention”, this method operates on the abstract feature maps (128×16×8) extracted by the CNN layers, focusing on recalibrating the complex, learned patterns themselves.

Finally, the Both-CNN Attention strategy (Figure 6c) incorporates SE-Blocks at both the input and output stages. This hybrid design leverages the benefits of both strategies: explicitly filtering noisy microphone pairs at the raw input level while simultaneously refining the high-level abstract features for precise regression. As detailed in the Section 6, this dual-stage attention mechanism yielded the most robust performance.

#### 4.3.2. Training Objectives and Loss Function

A significant challenge in acoustic DoA regression is the “boundary effect”. In a standard linear degree regression (0°–180°), the numerical distance between 0° and 180° is maximum, despite these angles being topologically adjacent in a circular domain. This discontinuity often leads to prediction distortions near the boundaries. Addressing this issue involves transforming the regression target from a scalar angle θ to a continuous vector representation on the unit circle: (sinθ,cosθ). This transformation eliminates the boundary discontinuity, allowing the network to learn the phase relationships smoothly. Consequently, the final output layer of the network consists of two neurons representing these sine and cosine values.

Optimization of this geometric representation utilizes a composite loss function combining prediction accuracy with geometric constraints. The total loss $L_{total}$ is defined as:

(2) $$L_{total}=\left(1-\alpha \right)\cdot L_{reg}+\alpha \cdot L_{norm}$$

In this equation, $L_{reg}$ represents the regression loss, quantifying the error between the predicted vector (${\hat{y}}_{sin},{\hat{y}}_{cos}$) and the ground truth ($y_{sin},y_{cos}$). Smooth L1 Loss (Huber Loss) was employed for $L_{reg}$ to prevent exploding gradients from outliers.

The term $L_{norm}$ serves as a geometric constraint to ensure the predicted vector lies on the unit circle (i.e., $\sin^{2}\theta +\cos^{2}\theta =1$). It is calculated as:

(3) $$L_{norm}=\frac{1}{N}\sum_{i=1}^{N}{\left(\sqrt{{{\hat{y}}^{2}}_{sin,i}+{{\hat{y}}^{2}}_{cos,i}+\epsilon }-1\right)}^{2}$$

The weighting factor α was empirically set to 0.1, assigning a 9:1 ratio between the regression loss and the geometric normalization loss. Optimization employed the Adam optimizer with an initial learning rate of $10^{-3}$. Additionally, a learning rate scheduler was implemented to dynamically decay the learning rate when the validation loss plateaus, thereby ensuring stable convergence and precise parameter tuning.

## 5. Simulations

Extensive simulations were conducted to validate the effectiveness of the proposed STHD feature and CNN regressor in a controlled, ideal environment. We utilized the Phased Array System Toolbox of MATLAB (Version 2024b) for this purpose. This section details the simulation setup, the data generation process involving noise injection and segmentation, and the experimental protocol used for evaluation.

### 5.1. Simulation Setup and Array Configuration

**Figure 7:**
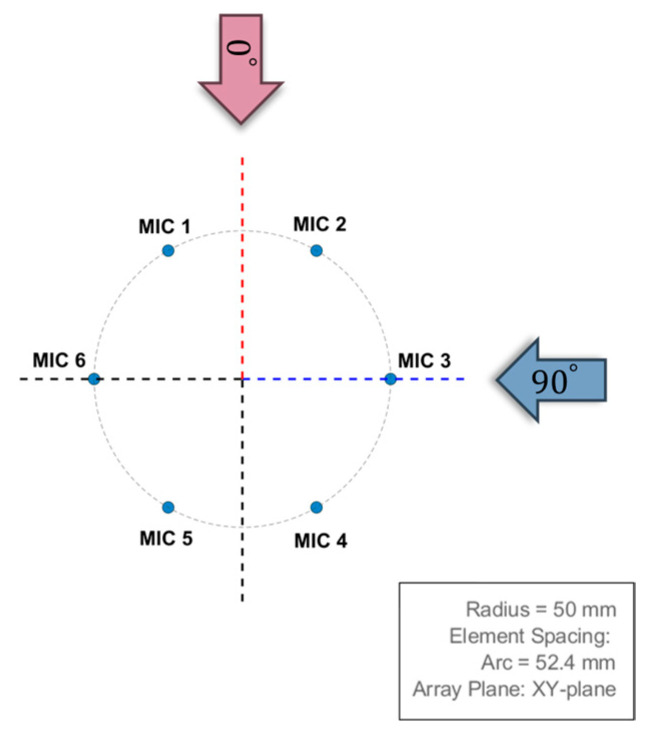
Configuration of the simulated 6-element Uniform Circular Array (UCA). The array features a 50 mm radius and includes a −120° rotation to align with the physical coordinate system of the ReSpeaker hardware used in experiments.

The simulation setup involved modeling a 6-element Uniform Circular Array (UCA) to replicate the geometry of the hardware used in practical experiments. The array elements were arranged on a circle with a radius of 50 mm centered at the origin (0, 0). However, the initial simulation model exhibited a −120° angular offset relative to the physical ReSpeaker 6-Mic Circular Array hardware. Consequently, to ensure precise consistency between the simulation and the real-world experimental environment, the virtual microphone positions were rotated by −120°. Figure 7 illustrates this aligned configuration, where the array geometry corresponds directly to the ReSpeaker hardware setup.

### 5.2. Data Generation and Preprocessing

Simulation of far-field sound sources covered azimuth angles ranging from 0° to 179° with a high resolution of 1°, resulting in 180 distinct target directions. Generation of 6-channel multi-channel audio signals involved applying appropriate time delays to the single-channel source signal, based on the specific array geometry and the Angle of Arrival (AoA). The detailed simulation parameters are summarized in Table 2.

Additive White Gaussian Noise (AWGN) was injected into the generated multi-channel signals to simulate realistic conditions and account for inherent sensor noise. Creation of separate datasets under clean (no noise) and noisy conditions, specifically with Signal-to-Noise Ratios (SNR) of 10 dB and 20 dB, enabled the evaluation of the model’s robustness against varying noise levels.

The generated 20 s continuous multi-channel signals were subsequently segmented into smaller windows suitable for model training. Each segment comprised 66,000 samples, corresponding to a duration of approximately 1.375 s at a sampling rate of 48 kHz. This segmentation process yielded 114 data instances per angle. Consequently, the total simulated dataset consisted of 20,520 data instances (180 angles × 114 instances/angle). Finally, the dataset was strictly partitioned into training (80%), validation (10%), and testing (10%) subsets.

### 5.3. Experimental Protocol and Evaluation Metric

Performance evaluation of the proposed regression framework utilized the dataset partitioned as described in Section 5.2. An ablation study comparing four distinct model configurations was conducted to rigorously validate the effectiveness of the attention mechanisms. The Simple CNN serves as a baseline model consisting of the 3-layer backbone without any attention blocks. The Pre-CNN Attention model incorporates the SE-Block directly at the input stage to weight STHD features. Additionally, the Post-CNN Attention variant applies the SE-Block to high-level features after the final convolutional layer. Finally, the Both-CNN Attention model integrates SE-Blocks at both the input and output stages to combine the benefits of both strategies.

Quantitative assessment of regression accuracy relied on two primary metrics: the Mean Absolute Error (MAE) and the Root Mean Square Error (RMSE). The MAE measures the average magnitude of angular errors, while the RMSE imposes heavier penalties on larger prediction deviations. These metrics are defined as follows:

(4) $$MAE=\frac{1}{N}\sum_{i=1}^{N}\left|y_{i}-{\hat{y}}_{i}\right|$$

(5) $$RMSE=\sqrt{\frac{1}{N}\sum_{i=1}^{N}{\left(y_{i}-{\hat{y}}_{i}\right)}^{2}}$$

In addition to these point estimates, we calculated the 95% Confidence Interval (CI) to assess the statistical reliability of the reported performance. The CI provides a range of values that is likely to contain the true mean error with a probability of 95%, offering a rigorously validated measure of the model’s stability.

**Figure 8:**
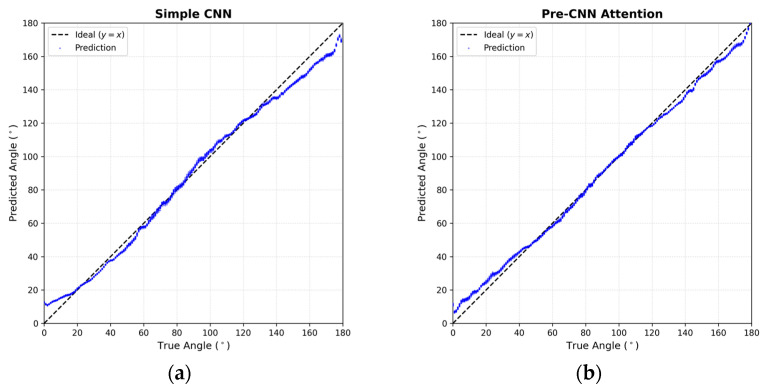

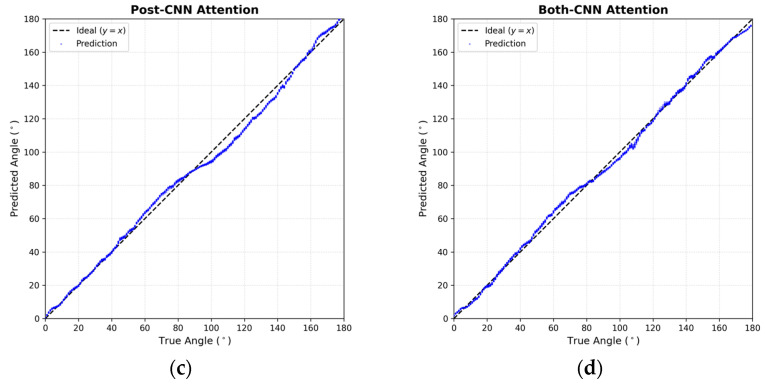
Scatter plots of ground truth vs. predicted DoA angles for the unseen testing dataset (2160 data instances) across different model architectures. (**a**) Simple CNN (Baseline), (**b**) Pre-CNN Attention, (**c**) Post-CNN Attention, and (**d**) Both-CNN Attention. The dashed line indicates the ideal prediction (y=x). The “Both-CNN Attention” model exhibits the tightest clustering, confirming superior regression accuracy.

Figure 8 presents the scatter plots of the predicted DoA versus the ground truth for the four comparative models, generated using the 2160 data instances from the testing dataset that were never seen during training. The red dashed line represents the ideal prediction (y=x). As observed in Figure 8a, the Simple CNN exhibits a relatively wider spread around the diagonal, indicating higher variance. Introduction of attention mechanisms (Figure 8b,c) visibly tightens this distribution. Notably, the Both-CNN Attention model in Figure 8d demonstrates the highest degree of linearity with the tightest clustering along the diagonal, suggesting that refining features at both the raw input and abstract levels effectively mitigates outliers.

Table 3 summarizes the numerical performance of these models. The results confirm that incorporating channel attention significantly reduces the angular error. The Pre-CNN Attention model achieved an MAE of 2.71°, outperforming both the Post-CNN Attention model (2.84°) and the Simple CNN (3.80°). This outcome supports the hypothesis that “Input-aware” recalibration is crucial for filtering reliable microphone pairs early in the process. Furthermore, the Both-CNN Attention model achieved the best overall performance with an MAE of 1.90° (95% CI: ±0.05°) and an RMSE of 2.30° (±0.06°), demonstrating that simultaneous optimization of input reliability and high-level spatial features yields the most robust DoA estimation.

To further validate the discriminative power of the proposed STHD feature, we conducted a comparative analysis against the widely used Generalized Cross-Correlation with Phase Transform (GCC-PHAT) [4] feature. For a fair comparison, the GCC-PHAT feature map was resized to match the input dimensions of our network, and the model architecture was fixed to the best-performing ‘Both-CNN Attention’ configuration. As shown in the last row of Table 3, the GCC-PHAT-based model yielded an MAE of 2.06° and an RMSE of 2.58°. In contrast, the proposed STHD feature achieved a superior MAE of 1.90° and an RMSE of 2.30°. This improvement indicates that the 2D cepstral representation of STHD effectively captures the temporal dynamics of the direct path more robustly than the correlation-based approach, particularly in resolving spatial ambiguities.

## 6. Results

### 6.1. Data Collection and Experimental Setup

Validation of the proposed model with real-world acoustic data involved experiments using the ReSpeaker 6-Mic Circular Array Kit connected to a Raspberry Pi 4. All acoustic experiments were performed in an anechoic chamber that conforms to ISO 3745 guidelines [28] to ensure measurement reliability and obtain a clean dataset free from environmental reverberations. The chamber is validated to support free-field operations in the 250 Hz–16 kHz range and hemi-free-field operations in the 1 kHz–16 kHz range (1/3 octave bands), ensuring a strictly controlled acoustic environment.

**Figure 9:**
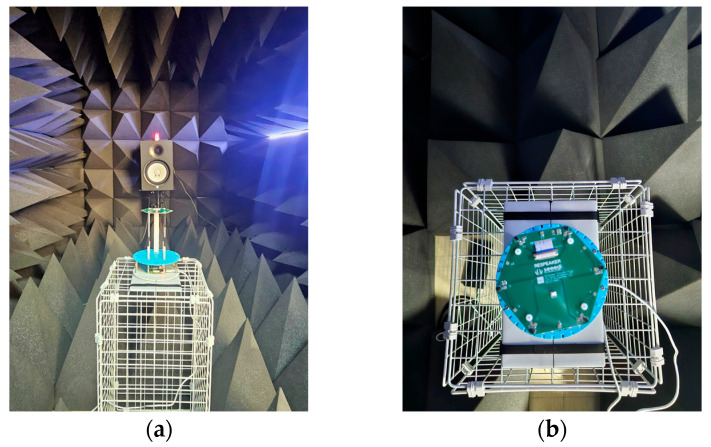
Experimental setup for real-world data collection. (**a**) The overall setup in the anechoic chamber, showing the ReSpeaker 6-Mic array aligned with the speaker configuration. (**b**) Close-up of the custom 3D-printed fixture. The structure rigidly connects the microphone array to the Raspberry Pi and features notches at 10° intervals. Measurements were taken at each notch and the intermediate midpoints to achieve a 5° resolution.

Figure 9 illustrates the experimental setup. Minimization of acoustic reflections from the support structure was achieved by mounting the microphone array on a minimal wire mesh stand. A custom-designed 3D-printed fixture was fabricated to rigidly couple the ReSpeaker array with the Raspberry Pi, ensuring precise vertical alignment and structural stability. As shown in Figure 9b, this fixture features a graduated disk with physical notches engraved at 10° intervals along its perimeter. To achieve a finer angular resolution of 5°, measurements were taken both at the notches and at the precise midpoints between them. The array was initially aligned with the sound source (a high-fidelity monitor speaker) using a laser level placed on top of the speaker, ensuring perfect linear alignment between the source and the center of the array.

Data collection covered 5° intervals ranging from 0° to 175°, resulting in 36 distinct target angles (0°,5°,…,175°). The recording process was controlled remotely via the Raspberry Pi to prevent noise or interference from human presence. For each angle, recording initiation was followed by the playback of a 20 s white noise sequence. A recording margin was added, resulting in a total file duration of 20.1 s per angle to ensure capture of the complete signal.

The collected raw audio files were processed using the same pipeline as the simulation. The 20 s continuous signals were segmented into 1.375 s windows. To maximize the size of the dataset and improve model robustness, we applied a sliding window technique with a 0.1 s stride during the segmentation process. The detailed specifications of the collected dataset are summarized in Table 4. Finally, the processed dataset was strictly partitioned into training, validation, and testing sets. The performance of the STHD-CNN model was evaluated using the Mean Absolute Error (MAE) and the Root Mean Square Error (RMSE) metrics to quantify the deviation between the predicted DoA and the ground truth angles.

To bridge the gap between the ideal simulation environment and the real-world acoustic conditions, we employed a transfer learning strategy. Specifically, the pre-trained weights from the simulation model were used as the initialization for the real-world model, which was then fine-tuned using the allocated 80% real-world training subset. This process allows the network to adapt to physical hardware characteristics while retaining the spatial feature representation learned from the large-scale simulation.

### 6.2. Regression Performance

Quantitative evaluation of the proposed STHD-CNN model efficacy relied on the Mean Absolute Error (MAE) and Root Mean Square Error (RMSE) metrics across the real-world experimental datasets. The MAE metric provides a direct interpretation of the average angular error in degrees.

Table 5 summarizes the performance on the unseen real-world test dataset. Despite the inevitable presence of hardware imperfections, microphone gain mismatches, and structural reflections inherent in physical setups, the proposed model demonstrated remarkable robustness. The Both-CNN Attention model achieved an MAE of 1.99° (95% CI: ±0.14°) and an RMSE of 2.38° (±0.14°). This performance is nearly identical to the simulation result (1.90°), confirming the excellent Sim-to-Real generalization capability of the proposed STHD features and network architecture.

A distinct contrast emerges when comparing the attention mechanisms. Unlike the simulation results where Pre-CNN Attention performed comparably to or better than Post-CNN Attention, in the real-world scenario, Pre-CNN exhibited higher errors (MAE 4.53°). In contrast, Post-CNN maintained high accuracy (MAE 2.33°). This suggests that in real-world environments containing physical noise, applying attention to high-level abstract features (Post-CNN) is safer than weighting raw input features directly, which may inadvertently amplify noise. However, the Both-CNN model successfully integrated the benefits of both strategies, achieving the lowest error rates.

**Figure 10:**
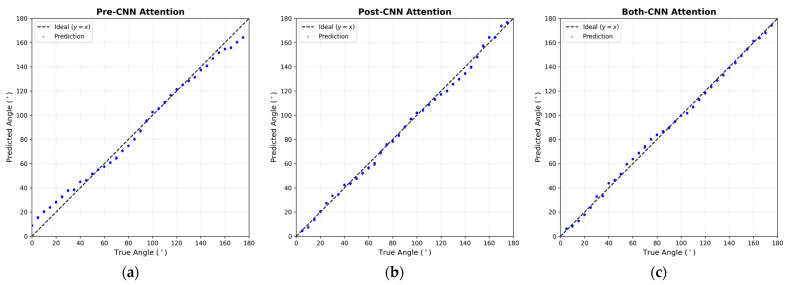
Scatter plots of ground truth vs. predicted DoA angles for the real-world testing dataset (360 data instances). (**a**) Pre-CNN Attention, (**b**) Post-CNN Attention, and (**c**) Both-CNN Attention. The dashed line indicates the ideal prediction (y=x). The “Both-CNN Attention” model exhibits the tightest clustering, verifying that the dual-stage attention mechanism is most effective for real-world acoustic data.

Figure 10 visualizes the regression performance for the 360 test data instances, where the x-axis represents the ground truth angles (collected at 5° intervals) and the y-axis represents the predicted angles. In Figure 10a (Pre-CNN), while the general linearity is preserved, a higher variance is observed compared to the other models. On the other hand, Figure 10b (Post-CNN) and Figure 10c (Both-CNN) show significantly tighter clustering around the ideal regression line (y=x). Specifically, the Both-CNN model (Figure 10c) effectively mitigates the boundary effects near 0° and 180° through the geometric loss function (sin,cos optimization), resulting in smooth and precise predictions across the entire angular range.

## 7. Conclusions

This paper presents a novel acoustic Direction of Arrival (DoA) estimation framework based on the Short-Time Homomorphic Deconvolution (STHD) feature. By extending the traditional 1D HD method into a 2D (Time vs. ToF) representation and utilizing all 15 microphone pair combinations from a 6-channel array, the temporal dynamics of the direct-path signal, critical for robust localization, were successfully captured. While traditional 1D HD methods condense the entire signal into a single static vector—masking transient acoustic events—the proposed STHD approach explicitly retains temporal resolution, allowing the system to distinguish stable direct paths from sporadic noise or reflections.

To effectively process these high-dimensional features, a lightweight 3-layer CNN regressor was designed. A key contribution of the proposed network architecture is the integration of a Dual-stage Channel Attention mechanism (Both-CNN Attention). This mechanism explicitly learns the reliability of each microphone pair at the input stage while simultaneously refining abstract spatial features at the output stage. Furthermore, addressing the boundary effect problem inherent in circular angle estimation was achieved by transforming the regression target into continuous vector coordinates $\left(\sin\theta ,\cos\theta \right)$ and applying a geometric constraint loss function.

The proposed system was rigorously validated through both MATLAB simulations and real-world experiments in an ISO-certified anechoic chamber. The results demonstrated that the Both-CNN model achieves high regression accuracy with a low Mean Absolute Error (MAE) of approximately 1.99° in both simulated environments and practical hardware setups. This successful generalization from simulation to real-world data confirms that the STHD feature, combined with the specialized dual-stage CNN architecture, provides a highly discriminative and stable representation for indoor sound source localization.

Future work will focus on extending this framework to more challenging real-world acoustic environments. While the primary objective of this study was to establish the fundamental validity and Sim-to-Real generalization of the proposed STHD feature under controlled conditions (i.e., white noise and anechoic chamber), subsequent research will rigorously test its robustness against non-stationary noise sources (e.g., speech babble, impulsive sounds) and varying reverberation times ($T_{60}$). Additionally, given the computational efficiency of the lightweight CNN, implementation and optimization of the system for real-time processing on edge devices (e.g., embedded systems or mobile robots) will be pursued to support dynamic indoor navigation tasks.

## 8. Patents

The contributions of this research have led to the publication of the following patents:

1. Park, Y.; Kang, S.; Keum, M.; Cho, K. Method, apparatus, system and computer program for sound source direction estimation, Republic of Korea Patent Application KR 10-2025-0025107, published 21 February 2025.
2. Park, Y.; Kang, S.; Keum, M.; Cho, K. Apparatus and method for collecting multi-channel audio training data, Republic of Korea Patent Application KR 10-2024-0025202, published 27 February 2024.

## Figures and Tables

**Table 1:** Specific parameters for Short-Time Homomorphic Deconvolution (STHD) implementation.

Parameter	Value	Parameter	Value
Sampling Rate ($F_{s}$)	48 kHz	Hop Size	256 samples
STFT Window	Hann	$\mathrm{FFT}\;\mathrm{Size}\;(N_{FFT}$)	512 points
Frame Length (N)	512 samples	Spectrum Type	Two-sided

**Table 2:** Simulation setup for data generation.

Parameter	Value	Parameter	Value
Number of receivers	6	Angle resolution	1°
Sampling frequency	48 kHz	Whole data set	20,520
Sound speed	340 m/s	Training set	80% of Whole
SNR	None, 10, 20	Validation set	10% of Whole
Angle range	0≤θ<180°	Testing set	10% of Whole

**Table 3:** Comparison of Mean Absolute Error (MAE) and Root Mean Square Error (RMSE) on the simulation testing dataset. The proposed STHD feature with Both-CNN Attention achieves the best performance. Values are presented as Mean ± 95% Confidence Interval.

Feature	Model	MAE (°)	RMSE (°)
STHD	Simple CNN	3.8012 ± 0.1268	4.8450 ± 0.1430
STHD	Pre-CNN Attention	2.7074 ± 0.0858	3.3850 ± 0.0967
STHD	Post-CNN Attention	2.8367 ± 0.0941	3.6076 ± 0.0914
STHD	**B** **oth-CNN Attention**	**1.8988 ± 0.0547**	**2.2984 ± 0.0577**
GCC-PHAT	Both-CNN Attention	2.0594 ± 0.0658	2.5831 ± 0.0711

**Table 4:** Experimental setup for data collection.

Parameter	Value	Parameter	Value
Angle range	0≤θ<180°	Training set	80% of Whole
Angle resolution	5°	Validation set	10% of Whole
Whole data set	3384	Testing set	10% of Whole

**Table 5:** Comparison of Mean Absolute Error (MAE) and RMSE on the real-world testing dataset. Values are presented as Mean ± 95% Confidence Interval.

Model	MAE (°)	RMSE (°)
Pre-CNN Attention	4.5318 ± 0.3403	5.5934 ± 0.3346
Post-CNN Attention	2.3375 ± 0.1678	2.8422 ± 0.1726
**B** **oth-CNN Attention**	**1.9883 ± 0.1357**	**2.3796 ± 0.1424**

## Data Availability

The acoustic data generated at various angles through MATLAB simulations, as well as the acoustic data collected at various angles in the anechoic chamber, are available from the corresponding author upon reasonable request.

## Abbreviations

The following abbreviations are used in this manuscript:

STHD	Short-Time Homomorphic Deconvolution
IPS	Indoor Positioning System
SSL	Sound Source Localization
DoA	Direction of Arrival
NLOS	Non-Line-of-Sight
TDOA	Time Difference in Arrival
GCC-PHAT	Generalized Cross-Correlation with Phase Transform
CNN	Convolutional Neural Network
HD	Homomorphic Deconvolution
ToF	Time-of-Flight
SCMR	Single-Channel Multiple-Receiver
AoA	Angle of Arrival
FFT	Fast Fourier Transform
RNN	Recurrent Neural Network
SE	Squeeze-and-Excitation
STFT	Short-Time Fourier Transform
IFFT	Inverse Fast Fourier Transform
UCA	Uniform Circular Array
FC	Fully Connected
AWGN	Additive White Gaussian Noise
SNR	Signal-to-Noise Ratio
MAE	Mean Absolute Error
RMSE	Root Mean Square Error
CI	Confidence Interval
